# Evaluation of Maximum Principal Stress, Von Mises Stress, and Deformation on Surrounding Mandibular Bone During Insertion of an Implant: A Three-Dimensional Finite Element Study

**DOI:** 10.7759/cureus.9430

**Published:** 2020-07-27

**Authors:** Bhavan Chand Yemineni, Jaideep Mahendra, Jigeesh Nasina, Little Mahendra, Lakshmi Shivasubramanian, Shareen Babu Perika

**Affiliations:** 1 Dental and Oral Surgery, Alluri Sitarama Raju Academy Of Medical Sciences College and Hospital, Eluru, IND; 2 Periodontics, Meenakshi Ammal Dental College and Hospital, Chennai, IND; 3 Operations & Information Technology, ICFAI Business School (IBS) Hyderabad - Institute of Chartered Financial Analysts of India (ICFAI) Foundation for Higher Education, Hyderabad, IND; 4 Periodontics, Maktoum Bin Hamdan Dental University College, Dubai, ARE; 5 Prosthodontics, Meenakshi Ammal Dental College and Hospital, Chennai, IND; 6 Periodontics, Care Dental College, Guntur, IND

**Keywords:** maximum principal stress, finite element, von mises, flexible deformity, implant success

## Abstract

Aim

The present study evaluated maximum principal stress, von Mises stress, and deformation on the mandible and surrounding structures during the insertion of an implant in various anatomical positions.

Materials and Methods

Finite element models of straight two-piece implants of 4.5 mm × 11.5 mm were modeled using Ansys software, v. 16.0 (Ansys, Inc., Houston, TX, USA). The mandibular model was derived through cone-beam computed tomography of a cadaveric mandible using Mimics software (Materialise NV, Leuven, Belgium). An osteotomy was performed at the first molar region, second premolar region, lateral incisor region, central incisor region, canine region, and second molar region that had varying bone densities. Implant insertion was simulated with a variable load of 1 - 180 Newton, which was applied axially downward with a rotational velocity of 30 - 120 rpm. Maximum principal stresses, von Mises stress distribution at the implant insertion site, and maximum deformation on the entire mandible were recorded during the insertion of the implants.

Results

Maximum principal stress was highest in the crestal area of the right first molar region and least in the middle third of the central incisor region during implant insertion. Von Mises stress in the mandible was highest in the right first molar region and the least in the lateral incisor region during implant insertion. The extent deformation was recorded on the x-axis, y-axis, and z-axis of the mandible. Deformation on the x-axis was highest at the crestal region of the canine and least for the lateral incisor. On the y-axis, deformation was highest at the symphysis region during implant insertion at the first molar region and the least at the condylar area during implant placement in the canine area. On the z-axis, the deformation was highest at the condylar region during implant insertion at the first molar region, and the least was observed in the symphysis region during implant placement in the second molar region.

Conclusion

When overall stress was considered, there is a direct correlation between stress and quality of bone. The highest maximum principal stress and von Mises stress were recorded during the placement of implants in posterior regions of the mandible, which suggests that the presence of dense cortical bone results in higher stress values. The maximum deformation was observed at different regions of the mandible, away from the site of implant insertion. The resultant stress and deformation exerted on the bone during placement of implants at different sites in the mandible varies, which could be detrimental factors in the longevity of the implant.

## Introduction

Osseointegration is exceptionally crucial for the longevity of a dental implant, in addition to the mechanical orientation of the implant with the surrounding bone. Primary stability is associated with this mechanical engagement [1-2]. Secondary stability also plays a significant role in longevity and is the consequence of stable bone regeneration and the remodeling process around the implant [3]. Micromovements can lead to deformations during the healing phase, which might disrupt the freshly formed biological bond between the bony tissue and dental implant [4-5]. Thus, the success of the implant highly depends on primary stability, for which a minimum insertion torque of 40 Newton centimetres (Ncm) has been recommended [6-7]. The bone type, bone quality, implant surface, and structure might play a significant role in stress distribution and deformation on the mandible during implant insertion, which could be crucial factors in determining the stability, and in turn, the longevity of the implant [8]. Von Mises stress is considered as a scalar value that is obtained from the stress exerted on the mandible, whereas principal stresses were obtained as a result of multi-axial stresses around the loading site. As principal stresses were evaluated from the exerted stress at a particular site, von Mises stress was also calculated directly from such stresses. Deformation in implant dentistry is described as the load applied in a framework for implant prosthesis that produces deformation energy in the system that causes flexion. If the framework consumes a maximum amount of deformation energy, there will be a reduction of the transmitted energy, which leads to a decrease in the stress in the structure [9].

The finite element analysis (FEA) method is widely used for analyzing models created using computer software. This method allows for the estimation of the biomechanical behaviour of the bone-implant interface and its components, as well as the simulation of the mechanical interaction, which could be difficult for in vivo experiments. FEA assists in applying different loads and determining displacement and stress levels within the tissues in vitro [10-11].

In the present study, three-dimensional (3D) explicit continuous nonlinear FEAs were performed. A constant angular velocity was applied to the implant, along with a vertical downward force. Through FEA, maximum principal stress at the implant insertion site, von Mises stress, and deformations on various areas of the mandible were evaluated.

## Materials and methods

An edentulous cadaveric mandible was obtained. A complete mandibular model was derived through cone-beam computed tomography of the cadaveric mandible by using Mimics software (Materialise NV, Leuven, Belgium) [12]. The bone constituted a spongy cancellous part surrounded by a cortical layer. The mandibular model was 3D, and the analyses were dynamic, continuous, and nonlinear.

Implant designing and osteotomy site preparation

**Figure 1:**
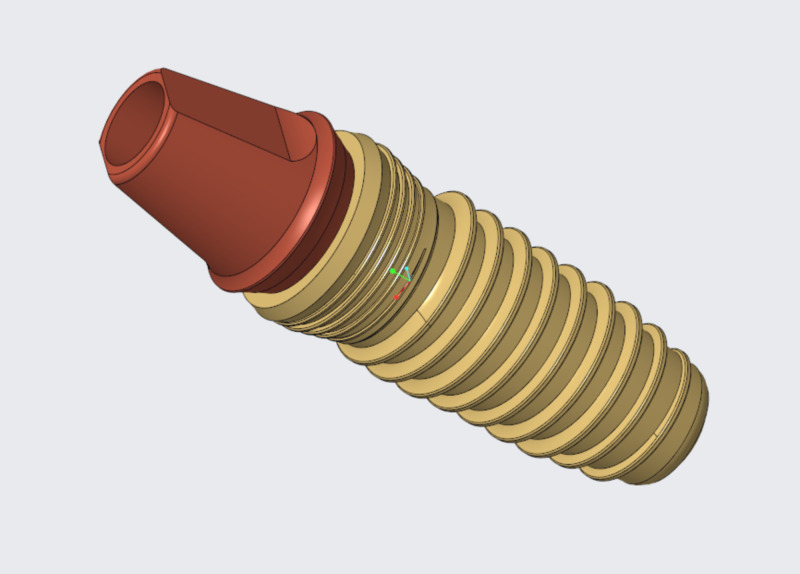
Finite element analysis (FEA) design of the dental implant (4.5 mm × 11.5 mm)

Six conical titanium implants of the dimensions of 4.5 x 11.5 mm connected to a screw-retained internal hexagonal abutment were designed (Figure 1).

The implant was made of a homogeneous, isotropic, and linearly elastic material. The physical properties, like Young's modulus and Poisson's ratio values of the implant components, were simulated to mesh the 3D finite element models of the dental implant [13].

Osteotomy sites of a length of 11 mm and a diameter of 4.25 mm were prepared in the first molar region, second premolar region, lateral incisor region, central incisor region, canine region, and second molar region of the mandible.

Implant insertion

During the insertion of an implant into the osteotomy site, the initial entrance phase was allied with the insertion of the restricted narrow apical portion of the implant into the cortical bone with a 2 mm thickness. During the thread penetration phase, the implant was inserted deep into the trabecular bone. Insertion torque values were controlled initially due to density variations between cortical and trabecular bones. During the second phase, torque values were expected to be higher due to the enhanced contact that results in more frictional forces acting as a resisting force. The osteotomy site simultaneously enlarged due to pressure exerted by the implant on the walls of the site. During the final phase of implant insertion, the neck of the implant was inserted into the bone; a decrease in the torque value may be observed.

Elements and nodes

After designing the models consisting of bone and implants, the solid geometries were exported for FEA. ANSYS software, version 16.0 (ANSYS, Inc., Houston, TX, USA) in STEP format was used for the study. A STEP file is a 3D model file formatted in *Standard for the Exchange of Product Data*, standardised exchange format. Following this, the tetrahedral elements formed the mesh. A convergence test of 10% determined the total number of control elements of the mesh for 370.345.

Finite element analysis

The simulation assumed a rigid implant, rotating at a constant rotational velocity of 30 - 120 rpm, with a downward, axially applied load that varied (arbitrarily) in the range of 1 - 180 Newton. During implant insertion, the maximum principal stress was recorded at the site of implant insertion. Von Mises stress distribution and maximum deformation were overall evaluated in the mandible.

## Results

Maximum principal stress

**Figure 2:**
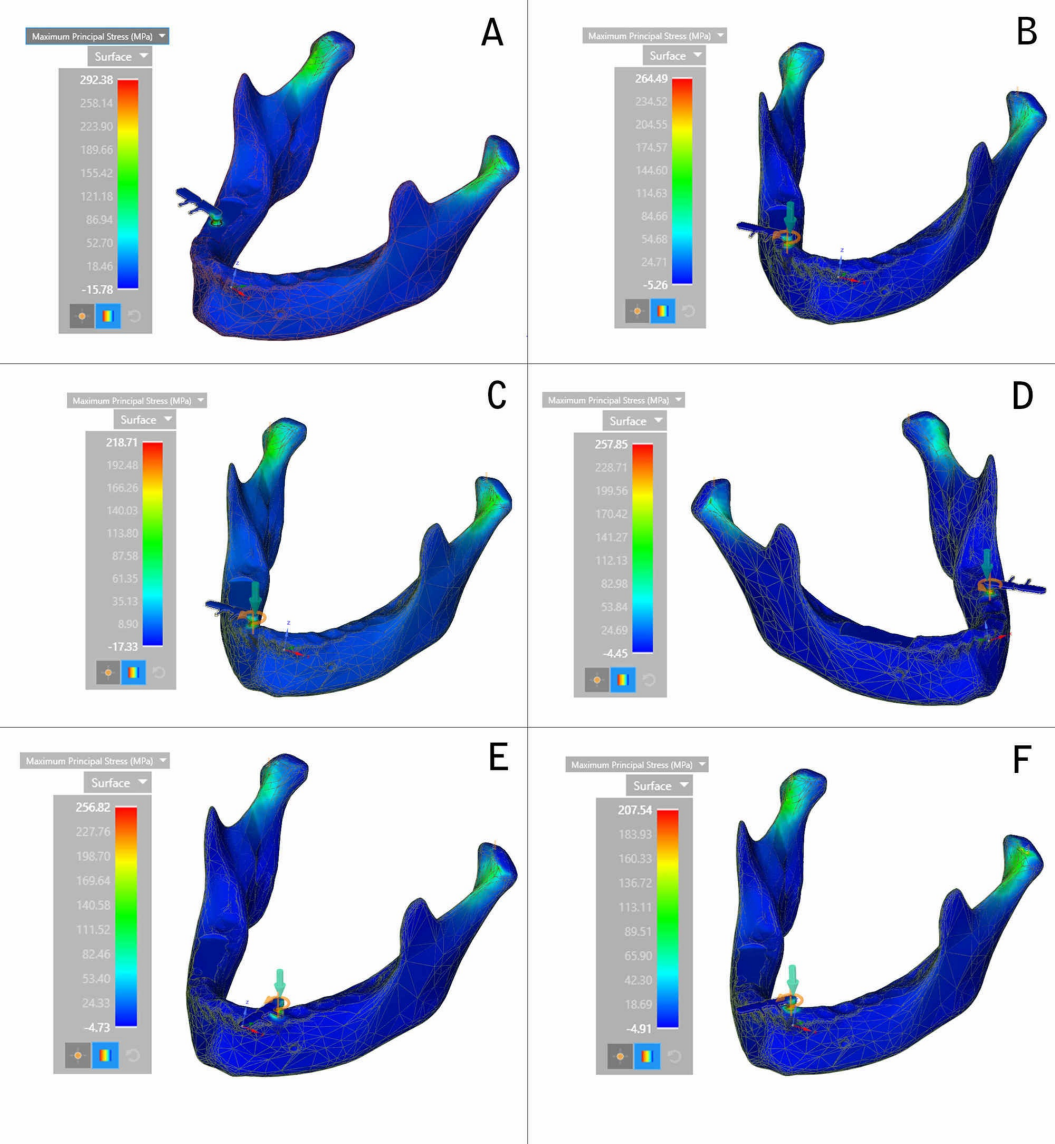
Maximum principal stress (A) Right first molar region; (B) Right second premolar region; (C) Lateral incisor region; (D) Left second molar region; (E) Left canine region; (F) Central incisor region

The maximum principal stress was recorded to be highest at the crestal region of the respective implants during insertion. The highest principal stress was recorded in the right first molar region and the least was in the case of the central incisor region. The decreasing order of maximum principal stress for all the six implants was the right first molar region, the right second premolar region, the left second molar region, the left canine region, the lateral incisor region, and the central incisor region (Figure 2).

von Mises stress

**Figure 3:**
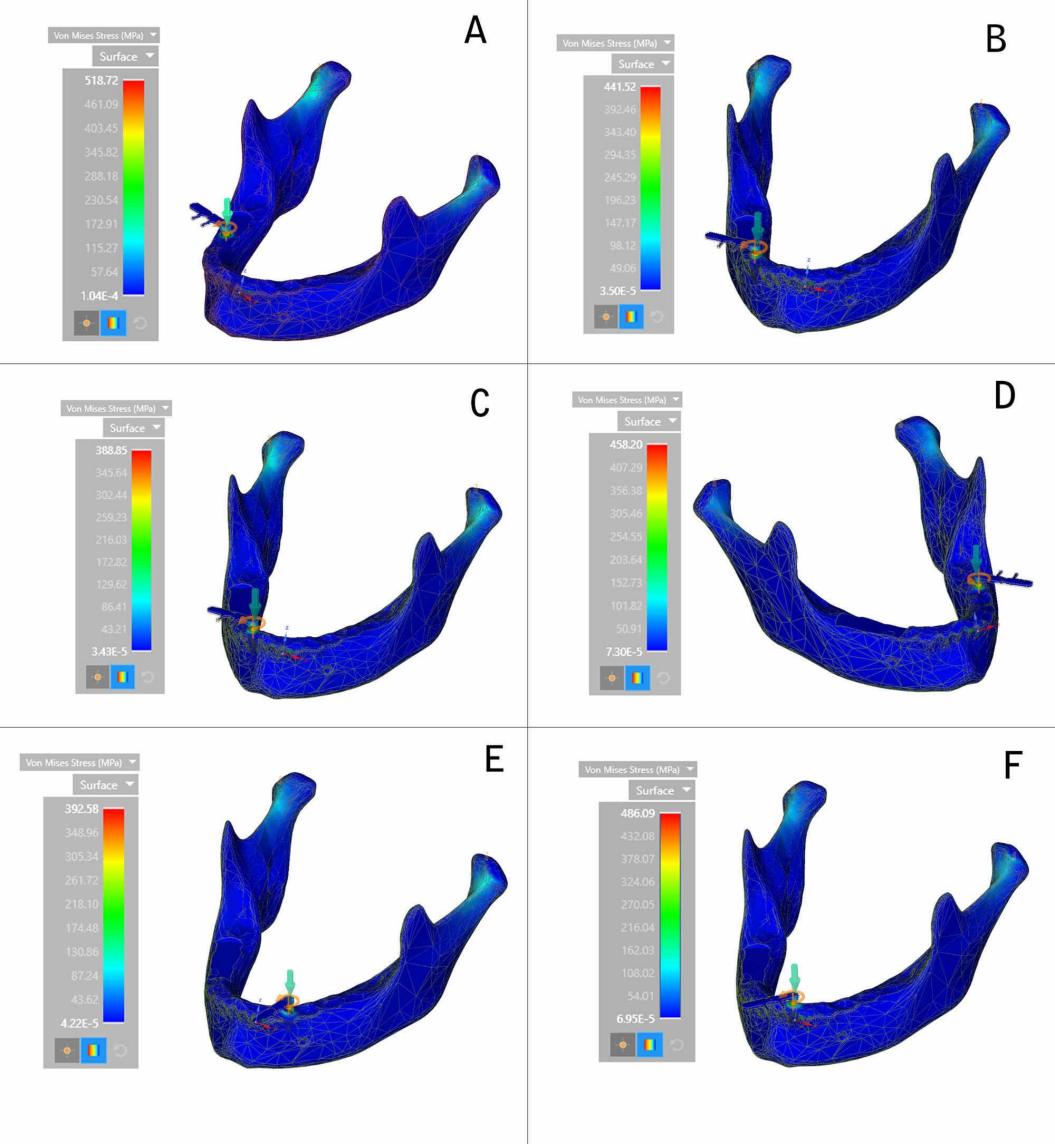
Maximum von Mises stress (calorimetric representation) (A) Right first molar region; (B) Right second premolar region; (C) Lateral incisor region; (D) Left second molar region; (E) Left canine region; (F) Central incisor region

The von Mises stress value was recorded on the whole of the mandible during the placement of all the six implants (Figure 3).

**Figure 4:**
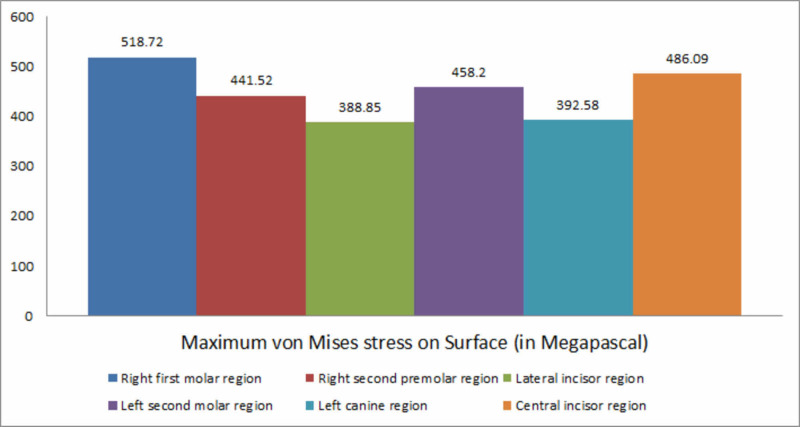
Maximum von Mises stress on the surface

The highest von Mises stress was observed in the region surrounding the right first molar region and the least was observed in the case of the lateral incisor region. The decreasing order of von Mises stress values in the mandible during implant placement is the right first molar region, the central incisor region, the left second molar region, the right second premolar region, the left canine region, and the lateral incisor region (Figure 4).

Deformation

**Figure 5:**
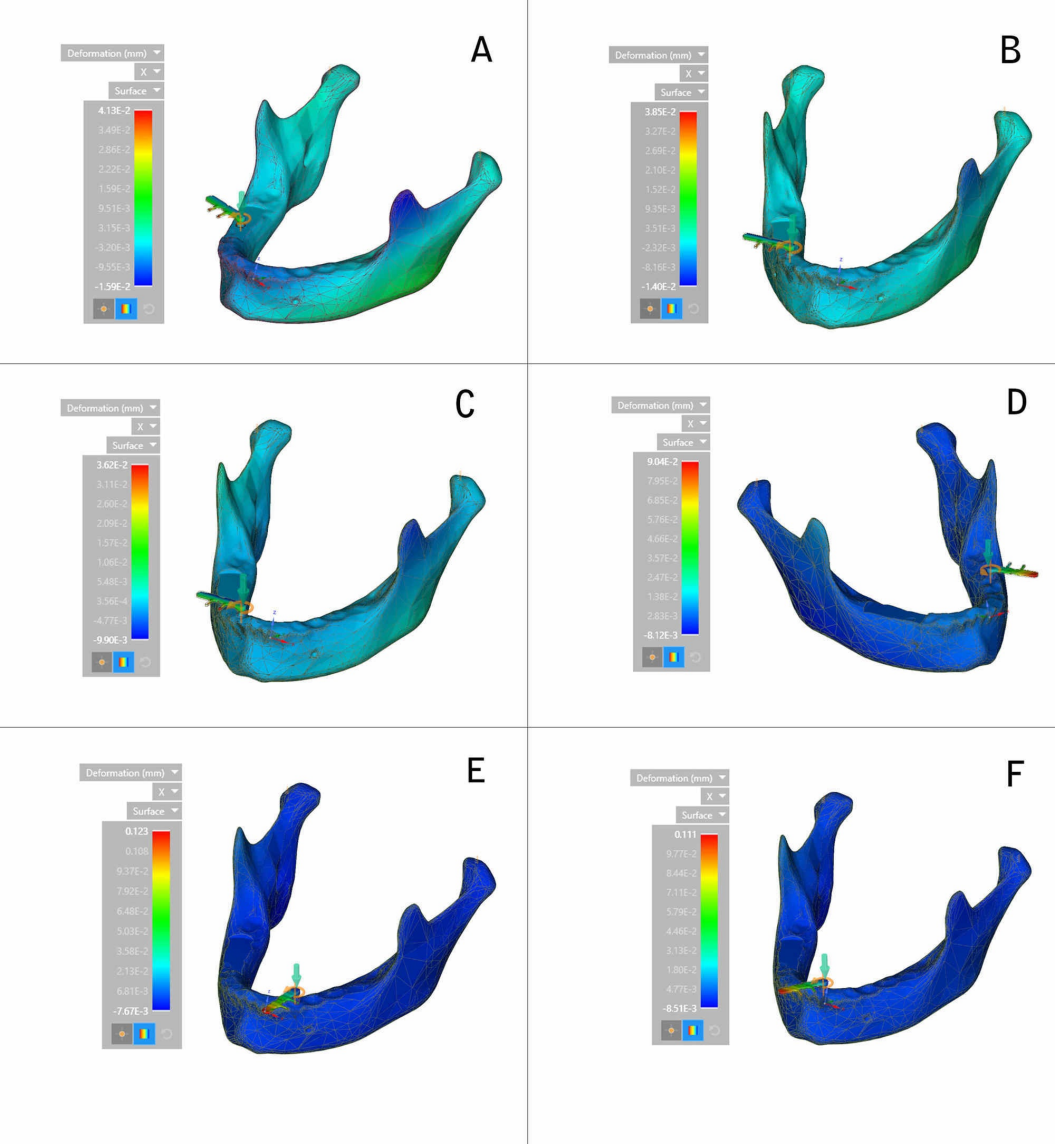
Deformation on the x-axis (calorimetric representation) (A) Right first molar region; (B) Right second premolar region; (C) Lateral incisor region; (D) Left second molar region; (E) Left canine region; (F) Central incisor region

Deformation was recorded for all the three planes of the mandible, i.e., x-axis, y-axis, and z-axis. In the horizontal plane or x-axis, the highest deformation was recorded at the angle of the mandible during the placement of an implant in the right first molar region (Figure 5).

**Figure 6:**
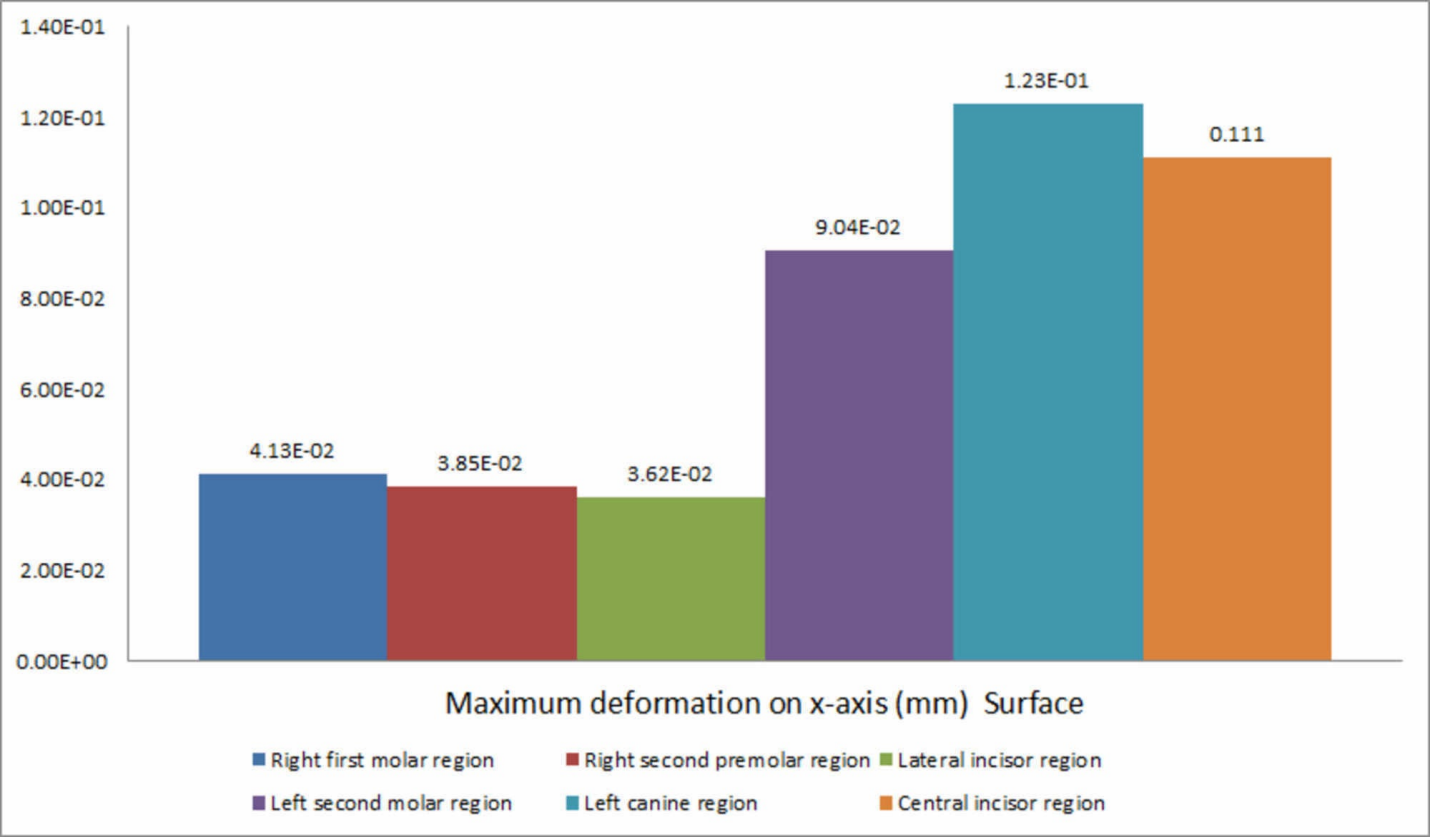
Deformation on the x-axis

The decreasing order of deformation on the x-axis for all the six implants placed was the right first molar region, the central incisor region, the left second molar region, the left canine region, the right second premolar region, and the lateral incisor region (Figure 6).

**Figure 7:**
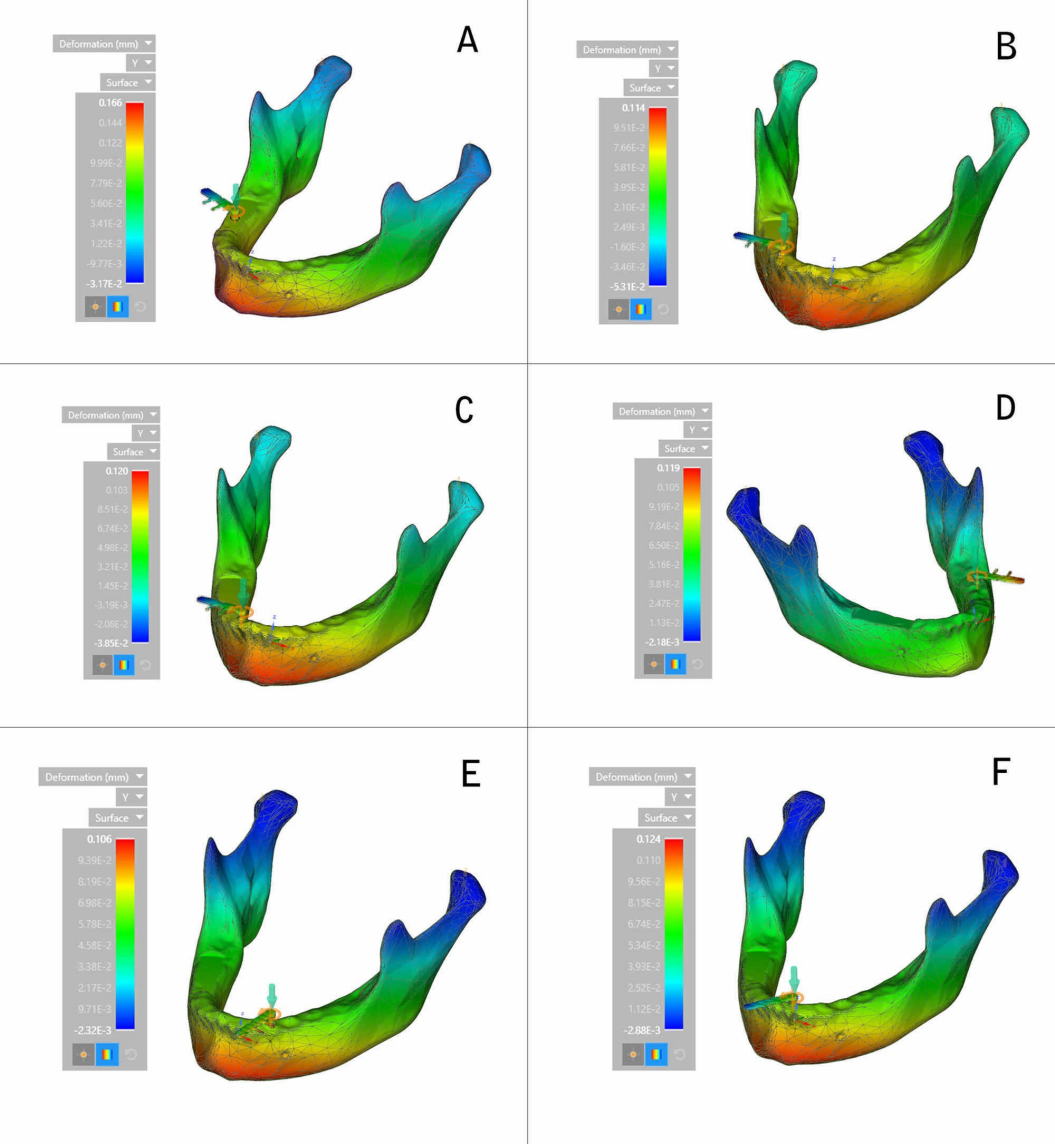
Deformation on the y-axis (calorimetric representation) (A) Right first molar region; (B) Right second premolar region; (C) Lateral incisor region; (D) Left second molar region; (E) Left canine region; (F) Central incisor region

In the vertical plane or y-axis, the highest deformation was recorded at the symphysis area during the placement of an implant in the right first molar region (0.166 mm) and the least in the left canine region (0.016 mm) (Figure 7).

**Figure 8:**
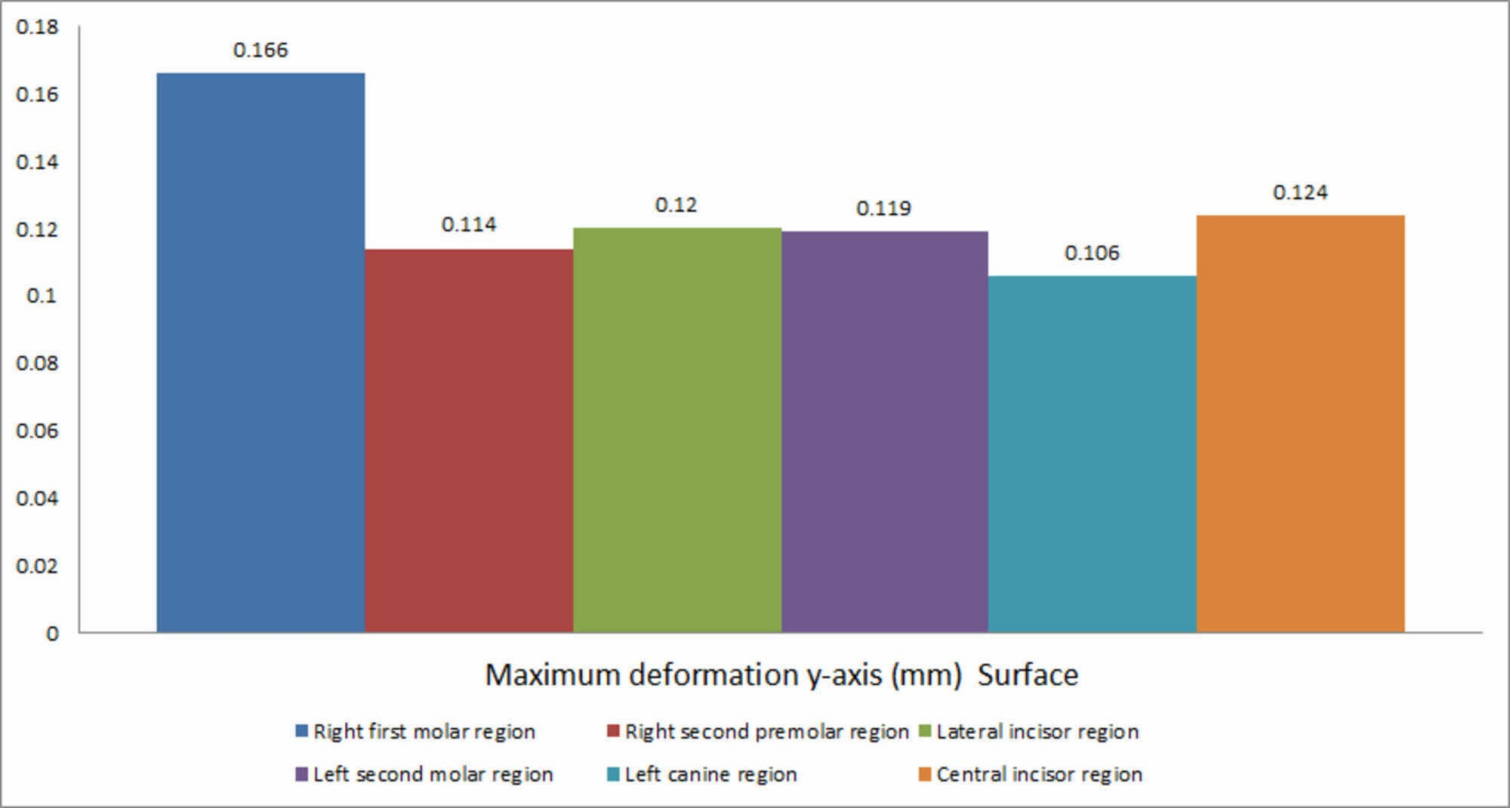
Deformation on the y-axis

The decreasing order of deformation on the y-axis for all the six implants placed was the right first molar region, the central incisor region, the lateral incisor region, the left second molar region, the right second premolar region, and the left canine region (Figure 8).

**Figure 9:**
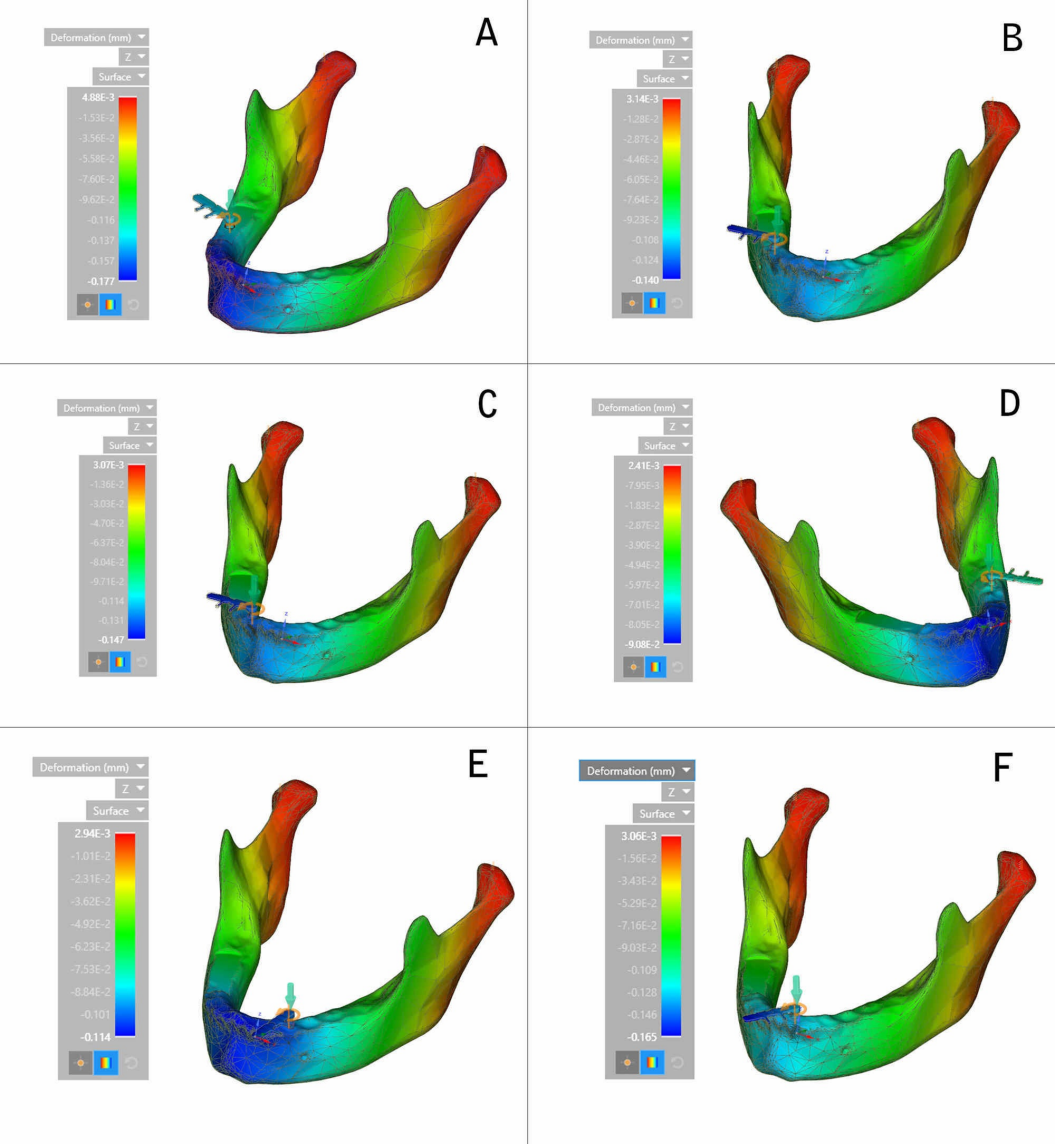
Deformation on the z-axis (calorimetric representation) (A) Right first molar region; (B) Right second premolar region; (C) Lateral incisor region; (D) Left second molar region; (E) Left canine region; (F) Central incisor region

In the oblique plane or z-axis, the highest deformation was recorded at the condylar region of the mandible during the placement of an implant in the right first molar region and the least at the left second molar region (Figure 9).

**Figure 10:**
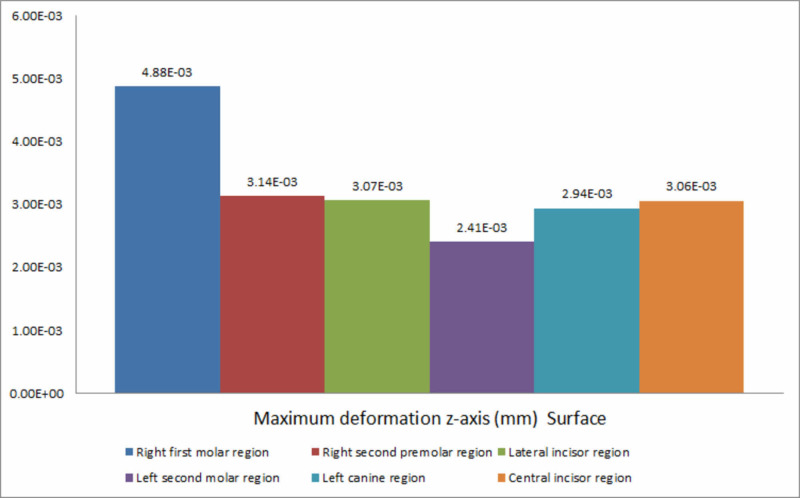
Deformation on the z-axis

The decreasing order of deformation on the z-axis for all the six implants placed was the right first molar region, the right second premolar region, the lateral incisor region, the central incisor region, the left canine region, and the left second molar region (Figure 10).

## Discussion

Distribution of stress and deformation on the bone during implant placement plays a pivotal role in the longevity of the implant. Excessive stress or deformation beyond the threshold might result in implant-related complications during and post-surgery. Hence, it is essential to consider these parameters during implant placement. The gold standard for evaluating stress distributed on the bone is the maximum principal stress, as well as the von Mises stress. Maximum principal stresses are the components of stresses when the basis of other stress tensors are zero and define the stress concentrated in a specific region. Von Mises stress, on the other hand, is a scalar quantity obtained from the stresses acting on any structure. It helps us to evaluate the yielding (or failure) of a ductile material. The idea of evaluating both stresses hold a critical point that von Mises stress is a measure of overall stress distributed on the mandible in all the axial planes. In contrast, maximum principal stress, as the definition says, is confined to stress exerted at a particular area during uniaxial loading. Maximum principal stress and von Mises stress are measured in units of megapascal (MPa).

Finite element analysis can be adapted as one of the finest modalities in analyzing the stress distribution and deformation. It is considered to be an appropriate method for evaluating stress exerted on the bone during insertion of the implant. Foreseeing how bone and implant components would behave, given the unique jaw anatomy for each clinical case, the quality of bone and the amount of occlusal force exerted on the prosthesis demands full comprehension of both mechanical and biologic events [14-15]. The understanding of the clinical findings and aggregation of factual data on stress distribution and deformation on the mandible, and other factors like implant loading, the bone-implant contact area may help us minimize the problems encountered in daily practice [16].

The present study aims to evaluate maximum principal stress, von Mises stress, and deformation on the mandible through FEA. The forces exerted around the implant, as well as the surrounding osseous tissues during implant insertion, were analyzed. The objectives of our study included the maximum principal stress concentrated around the osteotomy site, von Mises stress, and deformation on the entire mandible. Since there is a possibility of excess loads being transferred away from the area of implant insertion, von Mises stress and deformation were evaluated on the entire mandible dental implant insertion and can be performed manually or mechanically [8]. In our study, the implant was placed with a steady torque. The response and values of every element within the finite element model were assessed.

According to Misch and Tolstunov, the anterior mandible comprises the cortical bone with a density close to 1,250 Hounsfield units, followed by premolar and its posterior region of the mandible that are comprised of varying porous cortical and coarse trabecular to fine trabecular bone, respectively [17-18]. Hence, in our study, complete mandibular models were used to determine the effect of implant insertion on various structures of the mandible with varying densities. In a finite element model analysis, the behavior of the whole model is usually described by an enormously large set of equations that describe the behavior of individual elements joined together. Papavasiliou et al. and Kayabaşi et al. used the finite element model of a block bone for stress evaluation around the implant [13, 19]. However, to obtain dependable results on par with the clinical scenario, the number of elements and nodes must be 30,000 to 2,00,000, where increasing the number of elements provides more accurate results. Asmussen et al. and Maceri et al. employed finite element analysis in different restorative techniques [20-21]. Baggi et al. and Himmlová et al. also implemented the same to evaluate the influence of implant and prosthesis design on stress distribution [22-23]. In most of the above studies, the number of elements and nodes have either been limited or reduced outside the area of interest. Hence, in an effort to overcome the drawbacks of the previous studies, in our study model, we used specific regions that were separated into numerous small, simple blocks or elements, after which the structure was entirely made by joining the sets of elements together into a finite element model. On average, the model had 60,193 elements and 1,053,272 nodes. These numbers were well above the number of elements and nodes used in the majority of studies using FEA. Considering the geometrical complexity and appropriate discretization of the models, a 10% convergence was needed to ensure that the calibration of results should not be jeopardized. The consistency and connectivity of the mesh in the models demonstrated that this was a feasible model for the analysis of stress distribution employing 3D FEA. To enable contact pressure and shear movement between the implant and bone, a nonlinear face-to-face contact behavior was considered [24]. As a coefficient of friction of 0.45 had been proposed at the interface of metal surfaces and the bone by Viceconti et al. in their study, the same parameter was incorporated in our model [25]. 

The primary outcome variable of our study, the maximum principal stress, was highest in the crestal region of the right first molar region (292.38 MPa) and was least in the case the middle third of the central incisor region (207.54 MPa). In general, the highest concentration of maximum principal stress was observed at the neck region of all the implants during their insertion. In a study done by Guven et al., stress distribution was evaluated in periodontal and peri-implant bone tissues in 3- and 5-unit-dental and implant-supported zirconia restorations using FEA, wherein it was concluded that maximum principal stress was observed at the premolar and molar region for the implant-supported prosthetic model [26]. Türker et al. did the finite element stress analysis of applied forces to implants and supporting tissues using the all-on-four concept with different occlusal schemes and concluded that the highest stress on the implant was concentrated at the posterior region of the mandible when compared to the anterior region [27]. These were in accordance with the results of our present study showing that the maximum principal stress was concentrated at the posterior region of the mandible, suggesting that the presence of more cortical bone in the posterior aspect defines higher stress distribution in that area. 

In this study, the von Mises stress values indicated higher stress on the molar region (518.72 MPa) implant than on the incisor region implant. It could be because of dense cortical bone exerting more resisting force on the implant and, in turn, high von Mises stress values on the insertion of the implant in the molar region. Following the fact that the area of lesser bone density exerts comparatively lesser stress, the least von Mises stress was observed in the lateral incisor region (388.85 MPa). The varying bone densities in different regions of the mandible demonstrate a difference in overall stress that is exerted in a particular region. In their study, Skinner et al. evaluated the magnitude of maximum principal stress and von Mises stress during implantation of femoral implants and concluded that there was a direct correlation between bone density and maximum principal stress, von Mises stress [28]. Higher stress values were observed at the site of implantation where the bone density was higher or, in other words, in the presence of dense cortical bone. The results of our study are in accordance with the studies mentioned above, stating that a direct correlation exists between bone mineral density and resultant stresses.

Deformation during implant placement was observed to be highest on the vertical axis. This could be attributed to the vertical load exerted on the implant and resisting forces counteracting implant insertion. Within the respective axes, on the x-axis, a significant variation was observed between the extent of deformation exhibited during the insertion of implants in respective areas. Maximum deformation was observed in the canine region (0.123 mm), followed by the central incisor region (0.111 mm). This could be due to minimal bone thickness in the canine region and the increased probability of lateral forces being exerted on the implant, leading to deformation forces. On the y-axis, deformation was the highest in the right first molar region (0.166 mm) and the least in the left canine region (0.016 mm). On the z-axis, the highest deformation was recorded at the condylar region of the mandible during the insertion of the implant in the right first molar region, and the least was observed in the symphysis region during placement of the implant in the left second molar region.

The significant finding of this study is the understanding of the importance of density of bone as the outcome of the system; the built of osseous tissues, in turn, affects the biological and mechanical properties of the components surrounding the implants, such as the bone and resisting frictional forces [15]. The density values could influence the systemic characteristics during implant insertion. The implant needs to cross through the cortical bone initially and penetrate through the trabecular bone. To counter the higher stress exhibited by bone in cortical areas, certain improvisations might be needed in osteotomy procedures, which in turn could assist in the reduction of stress and deformation on the surrounding osseous tissues. At this crucial stage, modification of the osteotomy plays a significant role in implant longevity, which can be decided based on the values of reduced stress on the marginal bone. This decrease in stress could potentially reduce marginal bone loss. To sum it up, our study can lead to the analysis of stress on the marginal bone when modifications in osteotomy procedures are done, which could minimize the overall stress exerted on the bone and be beneficial in implant success. However, these results should not be taken as absolute, but they can relatively be applied as a comparison of the possible extent of stress distribution on bone and implant components during the insertion of an implant.

## Conclusions

High-stress values were reported in high-density areas. Minor variations in stress values were observed in different regions. As the stress on the surface marginal bone determines the longevity of the implant, higher stress can lead to marginal bone loss and implant failure. The present study emphasizes the use of a finite element analysis as an important part of the treatment planning before implant insertion as it would improve the longevity of the implant since, for the optimal clinical outcome, stress needs to be controlled during implant insertion.
